# Signature of Balancing Selection at the *MC1R* Gene in Kunming Dog Populations

**DOI:** 10.1371/journal.pone.0055469

**Published:** 2013-02-12

**Authors:** Guo-dong Wang, Lu-guang Cheng, Ruo-xi Fan, David M. Irwin, Shu-sheng Tang, Jian-guo Peng, Ya-ping Zhang

**Affiliations:** 1 State Key Laboratory of Genetic Resources and Evolution, Kunming Institute of Zoology, Chinese Academy of Sciences, Kunming, China; 2 Laboratory for Conservation and Utilization of Bio-resources, Yunnan University, Kunming, China; 3 Kunming Police Dog Base, Ministry of Public Security, Kunming, China; 4 Department of Laboratory Medicine and Pathobiology, University of Toronto, Ontario, Canada; 5 Banting and Best Diabetes Centre, University of Toronto, Ontario, Canada; Kunming Institute of Zoology, Chinese Academy of Sciences, China

## Abstract

Coat color in dog breeds is an excellent character for revealing the power of artificial selection, as it is extremely diverse and likely the result of recent domestication. Coat color is generated by melanocytes, which synthesize pheomelanin (a red or yellow pigment) or eumelanin (a black or brown pigment) through the pigment type-switching pathway, and is regulated by three genes in dogs: *MC1R* (melanocortin receptor 1), *CBD103* (β-defensin 103), and *ASIP* (agouti-signaling protein precursor). The genotypes of these three gene loci in dog breeds are associated with coat color pattern. Here, we resequenced these three gene loci in two Kunming dog populations and analyzed these sequences using population genetic approaches to identify evolutionary patterns that have occurred at these loci during the recent domestication and breeding of the Kunming dog. The analysis showed that *MC1R* undergoes balancing selection in both Kunming dog populations, and that the Fst value for *MC1R* indicates significant genetic differentiation across the two populations. In contrast, similar results were not observed for *CBD103* or *ASIP*. These results suggest that high heterozygosity and allelic differences at the *MC1R* locus may explain both the mixed color coat, of yellow and black, and the difference in coat colors in both Kunming dog populations.

## Introduction

Phenotypic diversity, including body size and coat color, among domestic dogs is overwhelming compared with that observed in their wild ancestors [1], [2], [3]. Morphological polymorphisms selected under domestication provide an excellent resource for unraveling the molecular basis of phenotypic diversity in domestic animals, and gives opportunities to examine the evolutionary patterns generated by artificial selection. The extremely diverse coat colors found in dog breeds is a good case for revealing the power of artificial selection, where the selection was imposed for human needs, and resulted in strong artificial selection and domestication bottlenecks [4].

Melanocytes synthesize pheomelanin (a red or yellow pigment) or eumelanin (a black or brown pigment) in mammals depending upon the genotype of several genes: *MC1R* (melanocortin receptor 1), *ASIP* (agouti-signaling protein precursor), and *CBD103* (β-defensin 103, also was known as K locus) [3], [5], [6]. MC1R is epistatic to both CBD103 and ASIP [3], [7]. Activated MC1R exclusively produces eumelanin and dominantly causes a uniform black coat, while inhibited MC1R exclusively produces pheomelanin and causes a uniform red or yellow coat [3]. CBD103 and ASIP bind to MC1R competitively and regulate the pigment type-switching pathway. *ASIP* encodes an extracellular inhibitory ligand of MC1R expressed on melanocytes [8], thus, gain-of-function mutations at ASIP yield the dominant inheritance of a yellow coat, while loss-of-function mutations at ASIP yield the recessive inheritance of a black coat. Conversely, CBD103 is a high affinity ligand of MC1R and competitively inhibits the ability of the ASIP protein to antagonize MC1R signaling [3]. Dogs carrying the dominant allele of *CBD103* have a black coat. Previous research has showed that mutations at these three genes can affect the coat color patterns in domesticated dogs [9]. For instance, a SINE insertion in *ASIP* causes the black-and-tan and saddle tan [10], a *CBD103* mutation causes a black coat color [3], and a specific *MC1R* allele causes the black mask pattern [11].

**Figure 1:**
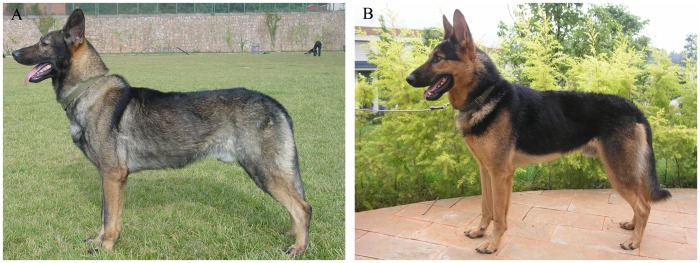
Photographs illustrating the differences in coat colors between the two Kunming dog populations. A) Wolf Black (WB) population. B) Back Black (BB) population. As shown, WB individuals have black and yellow all over their body, while BB individuals have black on their backs and yellow on their abdomen and limbs.

The Kunming dog breed was originally developed from the local hybrid dogs by crossing local native dogs with working dogs, such as the German shepherd, in the 1950s in Kunming, China. This hybrid has two stable populations, the Wolf Black (WB) and the Back Black (BB), which were derived from the Kunming dog breed during the past 16 years. Each of these two populations was bred separately by a random mating approach. Both populations have similar body size and behavior, differing only in coat color. Characteristically, males are 65 to 70 cm in height, with females being 60 to 65 cm, and they have body lengths that are slightly larger than their height. WB dogs have black and yellow all over their bodies, while dogs from the BB population have a black back and yellow abdomen and limbs (See Figure 1). These two stable Kunming dog populations provide an excellent resource to examine the evolutionary process that occurs to color genes under recent domestication process. Here, we resequenced and analyzed three coat-color-related genes: *MC1R*, *CBD103*, and *ASIP* in both Kunming dog populations using population genetic approaches. The results of these analyses show that: 1) Balancing selection occurs at the *MC1R* locus in both Kunming dog populations, but not at the *CBD103* or *ASIP* loci. 2) Significant differences occur between the WB and BB populations at the *MC1R* locus, but not at the *CBD103* or *ASIP* loci. This work reveals that the mixed color coat of Kunming dogs likely is due to balancing selection, and that the coat color difference between the two Kunming dog populations might be due to a divergence of the genotypes at the *MC1R* locus.

## Materials and Methods

### Population Samples and Sequence of *MC1R*, CBD103, and *ASIP*

The present study involves two populations of Kunming dogs. The WB population consisted of 44 individuals while the BB population consisted of 54 individuals. Figure 1 illustrates the differences in the coat colors of the two populations. Total genomic DNA was extracted from blood according to a standard phenol–chloroform extraction protocol. Sequences of 954-bp for *MC1R*, 1540-bp for *CBD103*, and 1920-bp for *ASIP* were amplified and resequenced using Sanger technology (Applied Biosystems). All PCR primers and resequencing primers were designed based on the dog reference genome (canfam2) (Table S1). Every nucleotide position of every individual was determined from both strands by at least one read each, and rare variants were confirmed by a second set of independent PCR products. Sequences from the dog reference genome were used as the reference sequence. All of the sequences obtained in the present study have been deposited into GenBank with accession numbers KC332295 - KC332882.

### Data Analysis

The haplotype phase of every individual was estimated with the PHASE 2.1 program [12], [13]. Linkage disequilibrium (LD) parameters (D′ and r^2^) and LD blocks were inferred using the Haploview program [14]. Summary of population genetic parameters, such as Watterson’s theta estimator (θ_W_), nucleotide diversity (θπ), haplotype diversity and population genetic analysis were calculated by DNAsp 5.10.01 [15]. Tajima’s D test [16] was performed using coalescent simulation under the assumption of no recombination across the genes, which is the conclusion of the Haploview analysis. Median-joining networks [17] were constructed by Network 4.5.1.6 to infer the haplotype genealogy (http://www.fluxus-engineering.com/). The program ms [18] was used to generate 10,000 independent replicated samples under the assumptions of the demographic history of the Kunming dog populations. We used the F statistic to evaluate the population differentiation according to the methods of [19] with the program GenepopV4 [20] which resulted in values between 0 (no differentiation) and 1 (complete differentiation). An exact G test was used for the statistical analysis. TMHMM 2.0 package (http://www.cbs.dtu.dk/services/TMHMM/) was used to predict the secondary structure of MC1R [21].

## Results

### Nucleotide Sequence Variation at *MC1R*, *CBD103*, and *ASIP*

To apply a population genetic approach, we resequenced the entire *MC1R* coding region, the complete *CBD103* coding region as well as one intron, and a part of *ASIP* in 44 Kunming dog individuals that have the WB coat color, and 54 Kunming dog individuals that have the BB coat color. A total of four SNPs (Single Nucleotide Polymorphism) were identified within the 954-bp *MC1R* sequence, all of which were non-synonymous substitutions (p.Ser90Gly, p.Ala105Thr, p.Pro159Gln, and p.Met264Val, nomenclature was described in http://www.hgvs.org/mutnomen/). Of the SNPs, three result in a change in amino acid properties, with the substitution p.Ser90Gly being from polar to nonpolar, and the substitutions p.Ala105Thr and p.Pro159Gln being from nonpolar to polar. All of these missense mutations had been previously identified in dogs [22], [23]. We identified 29 SNPs from the 1540-bp fragment of *CBD103*, and 9 SNPs from the 1920-bp fragment of *ASIP*. For *CBD103* and *ASIP* all of the SNPs are located in introns and untranslated regions (UTR) except for one synonymous mutation found in *CBD103* (g.1385C>T). The protein sequence encoded by *CBD103* in the Kunming dog is identical to the *k^y^* allele previously reported [3], [24].

### Haplotype Structure Analysis

**Figure 2:**
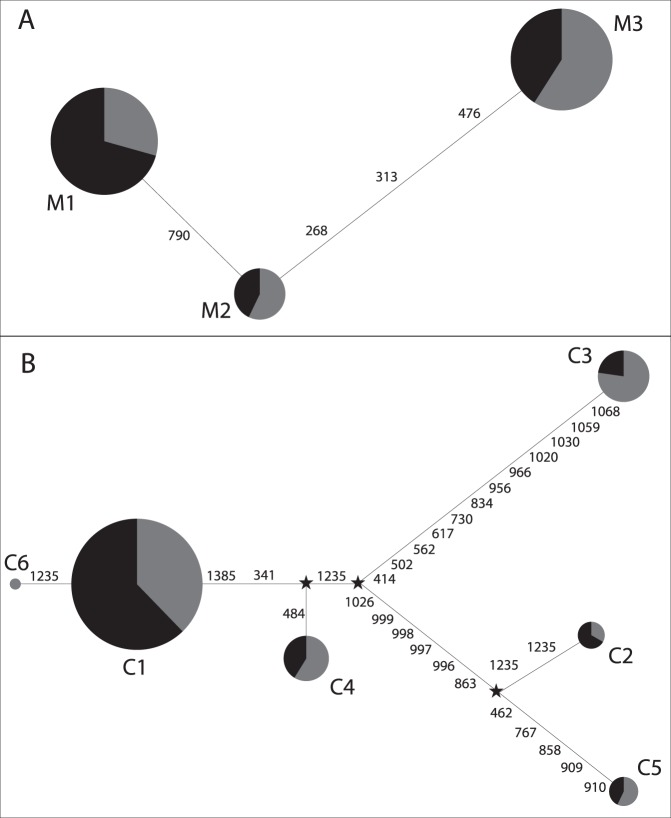
Median-joining networks showing the genealogy of *MC1R* (A) and *CBD103* (B) haplotypes in two Kunming dog populations. Each node in the network represents a different haplotype, and the size of each circle is proportional to the haplotype frequency. Circles are color-coded according to population (*Black:* BB population; *Grey:* WB population). The number of nucleotide differences between the haplotypes is shown on the branches of the network.

Haplotypes identified for the three gene loci are shown in tables S2 to S4. For *MC1R*, three haplotypes (M1, M2, and M3) were inferred in our populations, M1 was different from M3 for each SNP, while M2 was identical to M1 for the first 3 SNPs and identical to M3 at the last SNP. Interestingly, high heterozygosity was observed at the *MC1R* locus in both Kunming dog populations, compared with *ASIP* and *CBD103*, being 52.27% in the WB population and 48.15% in the BB population. Both Kunming dog populations had all three kinds of haplotypes. We identified 6 haplotypes (C1 to C6) in the resequenced region of *CBD103* and 2 haplotypes (A1 and A2) in the resequenced region of *ASIP*. LD analysis showed that each of the three genes were located in their own LD blocks (Figures S1–S3) in both populations. Further evidence for strong LD was provided by the four-gamete test [25], which reveals no recombination events for the three gene loci in our populations. We constructed median-joining networks [17] to study the genealogy of the haplotypes at the *MC1R* and *CBD103* loci. A median-joining network was not employed for *ASIP* as it has only two haplotypes. The topologies of the median-joining networks showed that *MC1R* presented two haplotypes (M1∶47%; M3∶42%) that are separated by relatively long-branch lengths, whereas *CBD103* presented only one major haplotype (C1), which represented 73% of the haplotypes (Figure 2). Divergent haplotypes at *MC1R* in each population might indicate the effect of balancing selection, or of demographic factors such as bottlenecks, population fusions, and/or founder effects.

### Evidence for Balancing Selection at *MC1R* in both Kunming Dog Populations

To determine whether balancing selection occurs at the *MC1R* locus in the two Kunming dog populations, we calculated the nucleotide diversity by means of θ_W_ [26] and θπ [27]. These two nucleotide diversity measures should be equal under a neutral model [16], however, our calculated θπ value was much larger than the θ_W_ value at the *MC1R* locus for both Kunming dog populations, with the θπ values being 2.43 and 2.35 times larger than the θ_W_ values in the WB and BB populations, respectively (see Table 1). The potential influence of artificial selection was further examined using the Tajima’s D test, which statistically compares the differences between θ_W_ and θπ [16]. At the *MC1R* locus, Tajima’s D value were significantly positive in both Kunming dog populations (one-tail test, see Table 1), although this may be underestimated as there are only four segregating sites [28], whereas no such pattern was observed for the *CBD103* and *ASIP* loci.

**Table 1:** Polymorphism statistics and neutral tests in the Kunming dog populations.

Population	Gene	Base pairs	N	S	Pi	Theta-W	Tajima’s D
Wolf Black	*MC1R*	954	44	4	0.00202	0.00083	2.90729**
	*CBD103*	1540	44	29	0.00431	0.00373	0.47848
	*ASIP*	1920	44	0	N/A	N/A	N/A
Back Black	*MC1R*	954	54	4	0.00188	0.0008	2.64503**
	*CBD103*	1540	54	29	0.00203	0.00358	−1.30205
	*ASIP*	1920	54	9	0.00057	0.00089	−0.88104

** Statistically significant at the 1% level.

**Figure 3:**
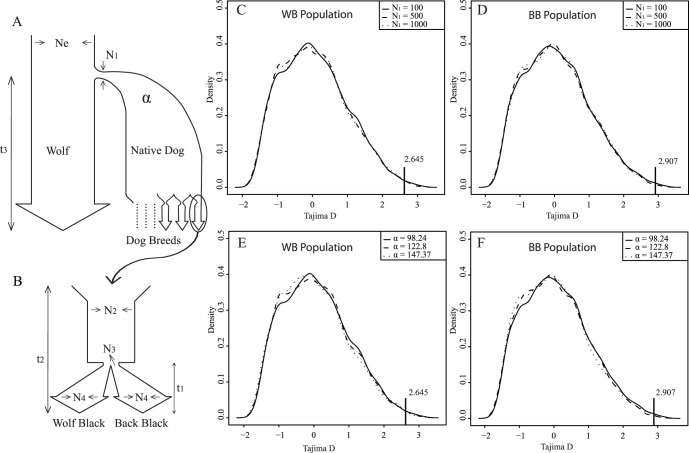
Standard coalescent simulations under the neutral model on the genealogy of the Kunming dogs. The demographic history of the Kunming dog populations can be divided into two stages: domestication and breeding periods. A) The domestication period started ∼15,000 years ago (t_3_). Ne represents the effective population size of the wolf population and N_1_ represents the size of the founder population of domesticated dogs. Domesticated dog underwent a period of population expansion, represented by the growth parameter α. B) The breeding period for the Kunming dog breeds started ∼70 years ago. N_2_ represents the size of the founder population of the Kunming dogs. Both Kunming dog breeds underwent a population size reduction (N3) followed by a period of population expansion (t_1_, N_4_). C) Standard coalescent simulations under different N_1_ (100, 500, and 1000) in the WB population. D) Standard coalescent simulations under different N_1_ (100, 500, and 1000) in the BB population. E) Standard coalescent simulations under different growth parameter α (98.24, 122.8, and 147.37) in the WB population. F) Standard coalescent simulations under growth parameter α (98.24, 122.8, and 147.37) in the BB population.

Significantly positive values for Tajima’s D can be generated by two processes: 1) balancing selection, and 2) demographic history. To examine whether demographic history played a role in the significant positive values for Tajima’s D at the *MC1R* locus, we did a demographic simulation that followed the demographic history of Kunming dogs for the three loci [18]. The demographic history of Kunming dog populations can be divided into two stages: a domestication period (Figure 3, A) which underwent a bottleneck early in domestication, and a breeding period which underwent a bottleneck during recent breed creation (Figure 3, B) [29]. According to previous research [30], [31], the dog was domesticated about 15,000 years (t_3_ ≈ 3750 generations if we assume 4 years per generation) from several hundred wolves (N_1_) whose effective population size was about 10,000 (*Ne* ≈ 10,000). The Kunming dog breed was created from domesticated dogs about 70 years ago (t_2_ ≈ 17 generations), and had a founder population of about 100 (N_2_ = 100). The WB and BB Kunming dog populations were generated about 16 years ago (t_1_ ≈ 4 generations), and the initial sizes of each population were about 15 (N_3_ = 15). At the time our samples were collected, each of the Kunming dog populations had a size of about 100 individuals (N_4_ = 100). We have assumed that the population underwent population expansion at t_1_ and t_3_ and was at a constant size at t_2_. All of the parameters are verifiable except N_1_ and the growth parameter α. N_1_ represents the size of the founder population, which might have been several hundred individuals [31]. Growth parameter α represents the growth rates in the domestication period, but has uncertainty, as we do not know the real number of domesticated dogs that existed in the 1950s. Since we did not have reliable estimates for N_1_ and α we therefore did multiple simulations using differing values for N_1_ (100, 500, and 1000) and α (98.24, 122.8, and 147.37; representing 5 million, 50 million, and 500 billion individuals in the 1950s, respectively). As shown in Figure 3, the results from all of the simulations show significantly positive Tajima’s D values for *MC1R* in both Kunming dog populations under the simulated demographic histories. For the different values of N_1_, 100, 500, and 1000, the *P* value of Tajima D test was 0.0016, 0.00071, and 0.0017 in the WB population, and was 0.0065, 0.0041, and 0.0067 in the BB population, respectively. For the different values of α 98.24, 122.8, and 147.37, the *P* value of Tajima D test was 0.0016, 0.00091, and 0.00071 in the WB population, and was 0.0065, 0.0039, and 0.0043 in the BB population, respectively. In contrast to *MC1R*, the Tajima’s D values for *CBD103* and *ASIP* were not significantly positive. These analyses suggest that *MC1R* has undergone balancing selection during the demographic history of both Kunming dog populations.

### *MC1R*, but not *CBD103* and *ASIP*, Show Population Differentiation

To determine whether population differentiation had occurred between the two Kunming dog populations, we analyzed the distribution of the haplotypes for the *MC1R* and *CBD103* loci. Interestingly, at the *MC1R* locus, M3 was the most abundant haplotype in the WB population, accounting for 55.68% of the sampled chromosomes, while M1 was the most abundant haplotype in the BB population, accounting for 60.19%. However, for *CBD103*, the same haplotype, C1, was most abundant in both Kunming dog populations, accounting for 61.36% and 82.41% in the WB and BB populations, respectively. The Fst value was calculated for measuring the degree of population differentiation between the WB and BB populations [19], [32] and the results are shown in Table 2. The Fst value for the *MC1R* locus across the two populations indicates significant genetic differentiation (Fst0.11, *P-value*0, exact G test), and each SNP of *MC1R* locus showed a significant signal for population differentiation, ranging from 0.101 (p.Ser90Gly, p.Ala105Thr, and p.Pro159Gln, *P-value* <0.001) to 0.146 (p.Met264Val, *P-value* <0.001). In contrast, the Fst value for the *CBD103* locus across the two populations did not show a similar result (Fst0.012, *P-value  = * 0.068), and none of the SNPs from *CBD103* showed a significant signal for population differentiation.

**Table 2:** Summary statistics of population structure.

Gene	Fst Value	Degree of freedom	Chi^2^	Genic differentiation
*MC1R*	0.1124	8	61.18412	0***
*CBD103*	0.0121	58	74.83048	0.0677

*** Statistically significant at the 0.1% level.

## Discussion

The pigmentation system of domestic dogs has undergone strong artificial selection yielding a high diversity of coat colors, with variation in the quantity, quality, and regional distributions. The pigment type-switching pathway, including the *MC1R*, *ASIP*, and *CBD103* gene loci, is a good model to research the influences of artificial selection. Here, we resequenced these three gene loci and applied population genetic approaches to identify the signature of artificial selection in two Kunming dog populations. For the *MC1R* locus, the resequenced region identified four previously known non-synonymous mutations, two of which have previously been reported to be associated with specific coat colors in dog populations: p.Ser90Gly is partially correlated with a black/brown coat [33], while having at least one copy of the p.Met264Val mutation, and not being homozygous for p.(Trp306*), was associated with the presence of a melanistic mask [11]. No nonsense mutations (p.(Trp306*)) were found in our populations, which is consistent with previous research as the homozygous p.(Trp306*) mutation causes a recessive pheomelanic phenotype [33], a trait that does not appear in either Kunming dog population. The protein sequence of *CBD103* coming from our population is identical to the *k^y^* allele previously reported, and not the 3-bp deletion allele (*CBD103^ΔG23^*) that is associated with a black coat [3], [24] and is in accordance with the observation that none of the Kunming dogs have a black coat. The resequenced region of *ASIP* did not include the region that encodes the substitution of R96C, a substitution that accounts for the recessive inheritance of a uniform black coat [34].

The divergent haplotypes and statistically significant positive Tajima’s D value suggests that *MC1R* might have undergone balancing selection or was influenced by demographic factors in both Kunming dog populations [35], [36], [37], [38]. To distinguish between these alternatives, we reconstructed the demographic history of the Kunming dog populations, which has been the characterized in previous research [29], [30], [31] and through records of breeding history. For each simulation of demographic history, we generated 10,000 independently replicated samples, calculated the nucleotide diversity, and conducted the Tajima’s D test. In brief, all simulation results strongly suggest that balancing selection, and not demographic history of either the domesticated dog or the Kunming breed, better explains the observed high nucleotide diversity at the *MC1R* locus, but not at the *CBD103* or *ASIP* loci. The most common reason for balancing selection is heterozygote advantage, a phenomena which has been well described for genes such as beta-hemoglobin [39], major histocompatibility complex-human leukocyte antigen (MHC-HLA) [40], and glucose-6-phosphate dehydrogenase (G6PD) [41]. Here, high heterozygosity at the *MC1R* locus in the Kunming dog populations is consistent with the mixture of yellow and black in the coat color of the Kunming dog.

Additional analyses showed that quite different haplotype distributions exist at the *MC1R* locus in the WB and BB populations, although both populations have a high heterozygosity caused by balancing selection. M3 was the main haplotype in the WB population while M1 was the main haplotype in the BB population. As shown in Table 2, clearly there is a significantly larger Fst value for the *MC1R* locus, compared to *CBD103*, revealing a significant difference between the WB and the BB populations. These results suggest that *MC1R* accounts for the coat color difference between these two populations and that the different compositions of the *MC1R* haplotypes might be responsible for the among-population differentiation in coat color patterns between the two Kunming dog populations. The results of TMHMM analysis showed that the p.Met264Val is located in the presumptive fourth-extracellular domain of *MC1R*, a region that contains important ligand binding sites [42], and that p.Ser90Gly and p.Ala105Thr are located in the second transmembrane domain, which is also involved in ligand binding [42], [43], [44], [45], [46], [47]. Variation in the sequences of these ligand-binding sites between the two Kunming dog populations may lead to differences in ligand-binding ability, and in turn, to differences in coat color.

## Supporting Information

Figure S1 **Linkage disequilibrium pattern of** ***MC1R*** **.**
(EPS)

Figure S2 **Linkage disequilibrium pattern for** ***CBD103*** **.**
(EPS)

Figure S3 **Linkage disequilibrium pattern for** ***ASIP*** **.**
(EPS)

Table S1 **Sequences of primer for PCR and resequencing of** ***MC1R*** **and** ***CBD103*** **, and** ***ASIP*** **.**
(DOC)

Table S2 **Summary of the differences in sequences, and haplotype distributions, for** ***MC1R*** **in the 98 Kunming dog individuals.**
(DOC)

Table S3 **Summary of the differences in sequences, and haplotype distributions, for** ***CBD103*** **in the 98 Kunming dog individuals.**
(DOC)

Table S4 **Summary of the differences in sequences, and haplotype distributions, for** ***ASIP*** **in the 98 Kunming dog individuals.**
(DOC)
