# Supplementary material for: Signature of Balancing Selection at the MC1R Gene in Kunming Dog Populations
Source: PLoS One. 2013 Feb 12;8(2):e55469. doi: 10.1371/journal.pone.0055469 (PMC3570536; doi:10.1371/journal.pone.0055469)
Supplement: Table S1 — Sequences of primer for PCR and resequencing of MC1R and CBD103 , and ASIP . (DOC) [file pone.0055469.s004.doc]

**Supplementary Table 1.** Primer Information for PCR and resequencing of *MC1R* and *CBD103*, and *ASIP*.

| Primer Name | Sequence (5'-->3') | Method |
| --- | --- | --- |
| Mc1r-2F | CAT TgC TgA gCT gAC ACT TgT ACA | PCR |
| Mc1r-2R | AgA ggg TCC ATg CAT CCA CC | PCR and Seq |
| mc1rS-1F | TgT ggA AAA TgT gCT ggT gg | Seq |
| mc1rS-2F | CAg CAT CgT CAC ACT CCC gC | Seq |
| mc1rS-3F | CCA CAC TCA CTA TCC TgC Tg | Seq |
| mc1rS-1R | gTC AAT gAT ggA gTT gCA gA | Seq |
| mc1rS-2R | gCT gAC AAg ACA AAg CAg gA | Seq |
| mc1rS-3R | CTC CAg CAC ATT gCT CAC gC | Seq |
| BetaD-1F | TTA TCA CgT ggT AAC AAC TgC C | PCR |
| BetaD-1R | gTC TgT ggg gTA AgA gAC ACT g | PCR and Seq |
| CBD103-S1 | gCA CCg ACC gCT CCT TAT T | Seq |
| CBD103-S2 | Agg gTC Agg TAg ggA gTT CAg T | Seq |
| CBD103-S4 | gAA Agg ACT ATg CAA CCA ACT g | Seq |
| CBD103-S5 | CTA CTT gCA gTC ACT AAg CTA g | Seq |
| CBD103-S6 | CAT ATT TgC gAT TCC TTC ACC C | Seq |
| CBD103-S7 | gAg ggg TgA Agg AAT CgC AA | Seq |
| CBD103-S8 | gAC CAT TTT ACC TAC TTg TgA g | Seq |
| CBD103-S10 | Agg AgC CCg ATg Tgg gAT TC | Seq |
| CBD103-S11 | ggg CTC Agg ggA AgA gTA A | Seq |
| CBD103-S12 | AgA ATA Agg AgC ggT Cgg T | Seq |
| CBD103-S13 | ATA AAC TTC CAg gAg gCA TT | Seq |
| CBD103-S14 | CTC TCT gTg TgA CTC TCA TA | Seq |
| CBD103-S15 | AAA CAA ACA ATT CCC TTg A | Seq |
| Ag-S-202 | gAg AAA ggg CTA CAg gAT gT | Seq |
| Ag-S-689 | CTg ggC ACT gTg AAg TgA gT | Seq |
| Ag-S-1076 | TgT TAT ATg gCA gAg TCA gg | Seq |
| Ag-S-1334 | TCC CCC ACC CTg AgA CTT CC | Seq |
| Ag-S-1572 | AAg ATT TTC CAg CCT TCC AT | Seq |
| Ag-A-1903 | CCT ggC Agg TTC TgT Tgg T | PCR and Seq |
| Ag-A-1645 | Tgg gCT TCT gTg ggg gTC Tg | Seq |
| Ag-A-1407 | CAg AAg gAg CAA AgC CAg AT | Seq |
| Ag-A-1146 | ACg ggA CCT gTg TTT TCT gTA | Seq |
| Ag-A-783 | gCC CTg CCA gTT AAg AAC T | Seq |
| Ag-A-329 | gCA ggg CTT TTC CAA ACC AT | Seq |
| Ag-S-598 | gAA AAC ATC Agg CAC ATT Ag | Seq |
| Ag-S-896 | TAg ATg TAT Cgg gAA gTA AC | PCR and Seq |
| Ag-A-229 | TAA gTT TgA CAT CCT gTA gC | Seq |
| Ag-A-573 | CAA CAC TgA AAg gAC TAA TT | Seq |
| Ag-A-916 | gAT AgT TAC TTC CCg ATA CA | PCR and Seq |
| Ag-A-1281 | TTA CTA gAC Tgg CTg CTg AC | Seq |
| Ag-A-1762 | CAA AAg AAT gAg TgA Tgg Tg | Seq |
| Ag-S-1764 | CTg AAT gCA ATg TCT CCT AA | Seq |
