# Supplementary material for: Signature of Balancing Selection at the MC1R Gene in Kunming Dog Populations
Source: PLoS One. 2013 Feb 12;8(2):e55469. doi: 10.1371/journal.pone.0055469 (PMC3570536; doi:10.1371/journal.pone.0055469)
Supplement: Table S2 — Summary of the differences in sequences, and haplotype distributions, for MC1R in the 98 Kunming dog individuals. (DOC) [file pone.0055469.s005.doc]

**Supplementary Table 2.** Summary of the differences in sequences, and haplotype distributions, for *MC1R* in 98 Kunming dog individuals.

| MC1R | Nucleotide positions | | | | Amino acid mutation | | | | Haplotype distribution | |
| --- | --- | --- | --- | --- | --- | --- | --- | --- | --- | --- |
|  | 268 | 313 | 476 | 790 | 90 | 105 | 159 | 264 | Wolf Black | Back Black |
| M1 | A | G | C | G | S | A | P | V | 27 | 65 |
| M2 | A | G | C | A | S | A | P | M | 12 | 9 |
| M3 | G | A | A | A | G | T | Q | M | 49 | 34 |
