# Supplementary material for: Signature of Balancing Selection at the MC1R Gene in Kunming Dog Populations
Source: PLoS One. 2013 Feb 12;8(2):e55469. doi: 10.1371/journal.pone.0055469 (PMC3570536; doi:10.1371/journal.pone.0055469)
Supplement: Table S3 — Summary of the differences in sequences, and haplotype distributions, for CBD103 in the 98 Kunming dog individuals. (DOC) [file pone.0055469.s006.doc]

**Supplementary Table 3.** Summary of the differences in sequences, and haplotype distributions, for *CBD103* in 98 Kunming dog individuals.

| CBD103 | Nucleotide positions | | | | | | | | | | | | | | | | | | | | | | | | | | | | | Haplotype distribution | |
| --- | --- | --- | --- | --- | --- | --- | --- | --- | --- | --- | --- | --- | --- | --- | --- | --- | --- | --- | --- | --- | --- | --- | --- | --- | --- | --- | --- | --- | --- | --- | --- |
|  | 3  4  1 | 4  1  4 | 4  6  2 | 4  8  4 | 5  0  2 | 5  6  2 | 6  1  7 | 7  3  0 | 7  6  7 | 8  3  4 | 8  5  8 | 8  6  3 | 9  0  9 | 9  1  0 | 9  3  1 | 9  5  6 | 9  6  6 | 9  6  7 | 9  9  6 | 9  9  7 | 9  9  8 | 9  9  9 | 1  0  2  0 | 1  0  2  6 | 1  0  3  0 | 1  0  5  9 | 1  2  3  5 | 1  2  6  8 | 1  3  8  5 | Wolf Black | Back Black |
| C1 | A | C | G | A | A | C | C | A | C | T | A | T | G | A | G | G | C | G | A | C | A | T | C | A | C | T | C | G | T | 54 | 89 |
| C2 | G | . | . | . | . | . | . | . | . | . | . | A | . | . | A | . | . | A | G | T | G | C | . | G | . | . | T | . | C | 2 | 4 |
| C3 | G | T | . | . | T | T | A | T | . | C | . | . | . | . | . | A | T | . | . | . | . | . | T | . | T | C | T | C | C | 17 | 5 |
| C4 | G | . | . | T | . | . | . | . | . | . | . | . | . | . | . | . | . | . | . | . | . | . | . | . | . | . | . | . | C | 10 | 7 |
| C5 | G | . | A | . | . | . | . | . | T | . | T | A | T | T | . | . | . | . | G | T | G | C | . | G | . | . | T | . | C | 4 | 3 |
| C6 | . | . | . | . | . | . | . | . | . | . | . | . | . | . | . | . | . | . | . | . | . | . | . | . | . | . | T | . | . | 1 | 0 |
