# Supplementary material for: Signature of Balancing Selection at the MC1R Gene in Kunming Dog Populations
Source: PLoS One. 2013 Feb 12;8(2):e55469. doi: 10.1371/journal.pone.0055469 (PMC3570536; doi:10.1371/journal.pone.0055469)
Supplement: Table S4 — Summary of the differences in sequences, and haplotype distributions, for ASIP in the 98 Kunming dog individuals. (DOC) [file pone.0055469.s007.doc]

**Supplementary Table 4.** Summary of the differences in sequences, and haplotype distributions, for *ASIP* in 98 Kunming dog individuals.

| ASIP | Nucleotide positions | | | | | | | | | Haplotype distribution | |
| --- | --- | --- | --- | --- | --- | --- | --- | --- | --- | --- | --- |
|  | 227 | 366 | 393 | 448 | 452 | 456 | 1573 | 1652 | 1884 | Wolf Black | Back Black |
| A1 | A | C | G | T | T | T | G | T | G | 88 | 101 |
| A2 | T | T | A | A | A | A | A | C | A | 0 | 7 |
